# Preliminary study on chronic granulomatous disease in Sri Lanka

**DOI:** 10.1186/s13223-018-0264-7

**Published:** 2018-09-17

**Authors:** Shalinda Jude Arjuna Fernando, Noorul Mifra Faiz, Shiroma Mangaika Handunnetti, Aruna Dharshan De Silva, Wasala Mudiyanselage Dhanushka Kumari Dasanayake, Geethani Devika Wickramasinghe, Rathnayake Mudiyanselage Chandima Hasanthi Karunatilake, Nilhan Rajiva de Silva

**Affiliations:** 1 Institute of Biochemistry, Molecular Biology & Biotechnology (IBMBB), University of Colombo, Colombo 03, Sri Lanka; 2 Genetech Research Institute, Colombo 08, Sri Lanka; 3 Department of Immunology, Medical Research Institute (MRI), Colombo 08, Sri Lanka

**Keywords:** Chronic granulomatous disease, NADPH oxidase, CYBB, NCF1, Mycobacterial infections, X-linked, Autosomal recessive, Sri Lanka

## Abstract

**Background:**

Chronic granulomatous disease (CGD) is a rare primary immunodeficiency of the phagocytic cells, which results in absent or diminished levels of microbicidal reactive oxygen species. The disease occurs due to germline mutations in the genes encoding the five subunits of NADPH oxidase complex. The present study is a pilot study to understand the clinical and genetic aspects of CGD in Sri Lanka.

**Methods:**

Clinical records of thirteen CGD patients were analysed and compared with similar studies performed in different countries and regions to identify patterns in demographics, clinical manifestations and infectious agents. Genomic DNA and cDNA were analysed in eight patients to identify mutations in *CYBB* and *NCF1* genes, thereby to ascertain the potential X-linked and autosomal recessive (AR) CGD patients.

**Results:**

The onset of symptoms in the patient cohort was very early (mean 4.6 months) compared to 20 months in India and 23.9 months in Latin America. Similarly, the age at diagnosis was lower (mean 1.6 years after birth) compared to other studies; 4.5 years in India and 6.1 years in Europe. Pulmonary manifestations were the most common (85%), followed by skin/subcutaneous infections (77%) and lymphadenopathy (62%). The death rate of local patients (38%) was higher than other countries (India 35%, Europe 20%). Majority (77%) were treated for tuberculosis at some point in life. Genetic analysis confirmed six out of eight patients as X-linked CGD cases with mutations in *CYBB* gene. A novel splice site mutation was identified in P-07 at position c.141+6 which resulted in the deletion of entire exon 2. Two siblings (P-05 and P-06) from consanguineous parents, were identified with AR-CGD based on the homozygous GT deletion mutation in *NCF1* gene.

**Conclusions:**

The clinical presentation, manifestations and genetic subtypes in the local cohort, appear to be comparable with global trends. Mycobacterial infections should be investigated and treated with more prominence. Effective treatment options are required to control the high mortality rate.

## Background

Chronic granulomatous disease (CGD) is a rare, inherited primary immunodeficiency which affects approximately 1/250,000 worldwide [1]. CGD is categorized into two main subtypes, viz. X-linked and autosomal recessive (AR), based on the mode of inheritance. Overall, about 65% of CGD cases worldwide are X-linked type while the remaining 35% are autosomal recessive CGD cases [2]. However, in certain regions with high degrees of consanguineous marriages, AR-CGD is more prevalent [3].

Chronic granulomatous disease is caused by a defect in phagocytic cells (neutrophils, macrophages, and monocytes) which fail to exhibit the ‘respiratory burst’, the rapid increase in oxidative metabolism following phagocytosis, thereby failing to effectively destroy invading pathogens. This is due to a germ line mutation in one of the five genes which code for the five subunits of the NADPH oxidase enzyme complex [1].

X-linked recessive CGD occurs due to mutations in the *CYBB* gene which encodes gp91*phox* subunit. Mutations in the genes encoding the subunits p47*phox*, p67*phox*, p40*phox*, and p22*phox* (*NCF1, NCF2, NCF4*, and *CYBA* respectively) are responsible for AR-CGD [4]. The most common form of AR-CGD accounting approximately 90% of all AR-CGD cases is due to mutations in *NCF1* gene. The predominant mutation found in these patients is a GT deletion in the GTGT repeat sequence at the beginning of exon 2 of *NCF1* [5].

The first CGD case was reported in Sri Lanka in 1999 [6]. There is a paucity of clinical data of CGD patients in Asian countries, especially South Asia. Large multi-centre studies have been carried out in Western countries, and CGD databases have been established in Europe, USA and Latin America. However, Western data might not be applicable for Asian countries due to differences in social practices (e.g. consanguineous marriages) and endemic infections. We report on the patients with CGD in Sri Lanka, and evaluate the clinical scenario and evolution of the disease in a developing country.

## Methods

### Patients

Ethics clearance was obtained from the Ethics Review Committee of the Medical Research Institute (No 05/2016 and 06/2016), and informed written consent was obtained from the parents of the patients.

Thirteen patients with CGD from 11 families were seen at Immunodeficiency Clinic at Medical Research Institute (MRI), Colombo Sri Lanka, from 2004 to December 2017. Their diagnoses were based on < 5% normal neutrophil activity with the nitroblue tetrazolium (NBT) reduction slide assay. Eleven out of thirteen patients were referred to the Immunodeficiency clinic owing to multiple recurrent infections. The two remaining patients were referred to the clinic, within days of birth due to recurrent infant deaths in the family. Clinical details of the patients, including details of deceased siblings, were obtained from the case records.

### Genomic DNA analysis

Three millilitres of blood was collected into tubes containing EDTA from eight CGD patients after informed written consent was obtained. Genomic DNA was extracted from whole blood stored in EDTA using commercially available QIAmp DNA Blood Mini Kit (QIAGEN, Hilden, Germany) following manufacturer’s protocol. Previously described [7] exon specific oligonucleotide primers for *NCF1* (2LB2 and 2RB2) were purchased from Integrated DNA Technologies (Iowa, USA). Thirteen sets of exon specific primers for the *CYBB* gene were kindly donated by Prof Yu-Lung Lau of University of Hong Kong. The amplified DNA fragments were subjected to bi-directional sequencing with BigDye^®^ Terminator v3.1 cycle sequencing kit and resolved with direct sequencing on an automated fluorescence sequencer, Applied Biosystems^®^ 3500 Dx Genetic Sequencer.

### cDNA analysis by RT-PCR

Human total leukocytes were isolated from EDTA-whole blood using dextran sedimentation method, which is based on differential centrifugation principle, as previously described [8]. Total RNA was extracted from separated leukocytes using TRIzol^®^ reagent as per the manufacturer’s guidelines. First-strand cDNA synthesis was performed from total RNA using random primers and the cDNA was immediately amplified into three overlapping fragments. The amplified products were subjected to bi-directional sequencing as described above.

## Results

### Demographics and family history

Eleven patients of the 13 analysed were males while the remaining two were females. The patients originated from 5 districts across the country. The patients comprised of all major ethnic groups of the country, 9 Sinhalese, 2 Tamil, and 2 Muslim patients. The ages of the patients ranged from 3 days to 14 years at the time of sampling. The participants of the present study were diagnosed between 2004 and 2017.

The thirteen CGD patients belonged to eleven families. P-01 and P-12 were brothers while P-05 and P-06 were brother and sister. Four families had consanguineous parents. Four patient families had reported 7 previous sibling deaths (Table 1). All of the sibling deaths occurred during infancy or very young age. In the family of P-01 three elder died due to sepsis. Although not diagnosed, two of the three siblings may have died of complications of CGD. The third sibling had been diagnosed with CGD (P-12), and was on prophylactic treatment. Furthermore, the mother of P-01 (and P-12) reported that three of her siblings passed away during infancy.

**Table 1:** Family history and clinical manifestations in CGD patients

Patient	Consanguinity	Sibling deaths	Age at manifestation	Clinical manifestation
P-01	Yes	C1 (M) Died at 4 months after febrile illness C2 (M) Died at 2 months. Post mortem examination revealed numerous caseating granulomata of lung and pleural tissue, consistent with bronchopneumonia due to tuberculosis (TB) (or *Mycobacterium bovis* following BCG) C3 (M, P-12) Diagnosed with CGD. Died at 3 years	1 month	Positive NBT. Diagnosed with CGD. On anti-microbial prophylaxis
P-02	No	No siblings	1½ months	Otitis media and meningitis
			2½ months	Septicaemia. Treated with meropenem for 7 days
			3 months	Left axillary lymph node abscess. Anti-TB category I and II for 1 year
			4 months	Right upper lobe pneumonia, otitis media
				Oral thrush twice while on IV antibiotics
			7 months	Positive NBT. Diagnosed with CGD. Prophylaxis commenced. Started anti-TB treatment
			2 years	Died following stem cell transplantation
P-03	No	C1 (M) Healthy	12 days	Nasal vestibulitis. Treated with IV Augmentin
			27 days	Multiple skin abscesses on hands and feet
				Right elbow joint osteomyelitis
			2 months	LRTI
				Positive NBT. Diagnosed with CGD. Prophylaxis commenced
			5 months	Skin abscesses on right forearm and left buttock
			7 months	Skin abscesses, pus discharge from BCG scar site, left axillary lymphadenopathy. Mantoux > 10 mm
P-04	No	C1 (F) Healthy C2 (M) Died at day 11 due to septicaemia	16 days	Fever, pyelonephritis, septicaemia, cellulitis, cervical lymphadenopathy, hepatosplenomegaly, and skin rash. Blood culture was positive for *B. cepacia* and *Candida albicans*
			43 days	Fever. Chest X ray revealed inflammatory changes. Lymph node biopsy revealed epithelioid cells and necrotizing inflammatory material. He was commenced on anti TB therapy
			2 months	Positive NBT. Diagnosed with CGD. Anti-microbial prophylaxis initiated
P-05	Yes	C1 (M) Died at 1 year 3 months after febrile illness	6 months	LRTI
			10 months	Middle mediastinal mass. Biopsy indicated caseous necrosis. Compatible with TB, treated with anti-TB therapy
			11 months	Pneumonia
			2 years	Recurrent oral ulcers
			2 years 7 months	Positive NBT. Diagnosed with CGD. Started on anti-microbial prophylaxis
			5 years 7 months	Oral thrush
P-06	Yes	Sibling of P-05	1 year 8 months	Urinary tract infection (culture positive)
				Multiple episodes of pneumonia, meningitis. Cervical lymphadenopathy
			4 years	Right cavitatory pneumonia, failure to thrive
			6 years	Left lower eye lid abscess and cellulitis
				*Pseudomonas aeruginosa* septicaemia
				Ecthyma gangrenosum, parotitis
				Positive NBT. Diagnosed with CGD. Anti-microbial prophylaxis initiated
			10 years	LRTI. Sputum negative TB. Anti-TB therapy category I and later II commenced
			11 years	HRCT Chest—early parenchymal and interstitial lung fibrosis mainly affecting upper lobes, and bronchiectasis of lower lobes
			14 years	Bronchopneumonia, blood culture revealed *C. parapsilosis*
				LRTI
			15 years	LRTI, abscesses
P-07	No	No siblings	1 months	Poorly resolving pneumonia, high fever spikes
			2½ months	Multiple skin abscesses
				Positive NBT. Diagnosed with CGD. On anti-microbial prophylaxis
			4 months	Abscesses occurred in scrotum, cheek and liver
			1 year 2 months	Blood stained stools. Right inguinal lymphadenopathy
			3 years	Anal fissure
				Inguinal lymphadenopathy. Excision biopsy revealed granulomata and central suppurative necrosis
			3½ years	Poorly resolving pneumonia (right middle lobe and lower lobe consolidation). Mantoux 18 mm
				Anti-TB category I commenced
			4½ years	Middle/left lobe pneumonia
			5 years	Pustules on the face. On itraconazole and cotrimoxazole prophylaxis
P-08	No	C2 (M) Healthy	7 months	Dysentery
			8½ months	LRTI
			1 year 9 months	Fever of unknown origin (21 days)
			3½ years	Mediastinal mass. Biopsy revealed extensive caseous necrosis. Acid fast bacilli not seen. Mantoux negative. Anti-TB therapy category I commenced (7 months)
			3 years 9 months	Left lower lobe pneumonia
			4 years	Right middle lobe pneumonia, lymphadenopathy
			4 years 5 months	Anaemia, hepatosplenomegaly. Treated with Iron (7 months)
			4 years 8 months	Fever, erythematous pustular rashes on lower limbs. Bone marrow showed increased lymphoplasmacytic activity
				Perihilar lymphadenopathy. TB culture, TB-PCR negative. Query—relapse of TB. Started on anti-TB therapy category II (7 months)
			5½ years	Fever for 1 month while on category II anti-TB therapy. Multiple areas of consolidation in lungs, mediastinal lymphadenopathy and hepatosplenomegaly. CT thorax guided biopsy—granulomatous inflammation suggestive of TB. Acute bronchopneumonia
			6 years 4 months	Positive NBT. Diagnosed with CGD. Prophylaxis commenced
P-09	Yes	C1 (M) Healthy C2 (F) Healthy	3 days	Fever, mild jaundice. Treated with IV antibiotics
			2 months	Meningitis, bronchopneumonia. Treated with IV antibiotics
			3 months	LRTI. Treated with IV cefotaxime
			4 months	LRTI
			8 months	Fever, meningitis. Mantoux 26 mm. Anti-TB therapy started
			11 months	Positive NBT. Diagnosed with CGD. Prophylaxis commenced
			2 years	Patient expired
P-10	No	C1 (M) Died at 1½ years following possible pneumonia C2 (F) Healthy C3 (M) Died at 1 year 2 months following possible pneumonia C4 (M) Healthy C5 (F) Healthy	1 year 1 month	Right middle lobe pneumonia, lung abscess
				Positive NBT. Diagnosed with CGD. Prophylaxis commenced
			4 years	LRTI and perineal abscess
			6 years	Bronchopneumonia
			7 years	Skin abscesses over right knee joint
			9 years	Cystitis, splenomegaly
			10 years	Patient expired
P-11	Yes	No siblings	1½ months	Severe failure to thrive, bilateral granulomatous cervical lymphadenitis. Defaulted anti-TB treatment
			4 months	Poor weight gain. Tonic convulsions, sepsis, hepatosplenomegaly. TB meningitis suspected. PCR of gastric aspirate for mycobacteria (GeneXpert) negative. Anti-TB therapy commenced
			6 months	Positive NBT. Diagnosed with CGD. Prophylaxis commenced
			1 year	Patient expired
P-12	Yes	Elder sibling of P-01	2 months	Admitted with febrile illness, and treated for sepsis with IV antibiotics for 21 days. Itraconazole and cotrimoxazole prophylaxis
			8 months	Skin abscess after DTP dose 3, liver abscess, fever, iron deficiency anaemia. IV antibiotics for 1 week. Liver abscess drained
			11 months	Positive NBT. Diagnosed with CGD
			2 years 3 months	Measles, fever, respiratory features, abdominal distension. Treated with IV antibiotics
				Mesenteric and paracentric lymphadenopathy. Anti-TB therapy commenced. Pus culture from abdominal wall abscess positive for MRSA
			3 years 3 months	Liver abscess, lymphadenopathy. Patient expired due to possible TB compilations
P-13	No	No	4 months	Skin abscesses
				Poor wound healing
			6 months	Bronchiolitis
			7 months	Pyrexia
				*Pseudomonas aerugenosa* isolated from wound swab
				Positive NBT. Diagnosed with CGD. Prophylaxis commenced

### Clinical course

Eleven patients were referred to MRI due to recurrent unresolving infections while P-01 and P-12 (siblings) were referred owing to their family history. The mean age at onset of disease among the patients is 4.6 ± 5.6 months. Majority of the patients (58%) manifested symptoms as early as 2 months since birth, out of which 3 patients developed symptoms during the first 2 weeks. In X-linked CGD patients, the mean age at onset was 1.5 ± 1.3 months and in AR-CGD patients 12 ± 8.5 months.

The mean age at diagnosis for the entire cohort was 18.6 ± 25.8 months. Most patients (69%) were diagnosed in infancy. The mean age at diagnosis was 4.6 ± 3.7 months in X-linked CGD patients and 51.5 ± 29.0 months in AR-CGD patients. The mean diagnostic delay irrespective of subtype was 15.5 ± 22.8 months.

Five out of 13 patients (38%) have died, at an average of 3.65 ± 3.63 years. Only 2 of the deceased patients (P-02, P-12) are known to have X-linked CGD. P-02 died following stem cell transplantation.

### Clinical manifestations

Respiratory tract was the most prevalent site of clinical manifestations. Eleven patients (85%) had respiratory tract infections, including bronchopneumonia (69%). Six patients (46%) had more than one episode of pneumonia. One patient had bronchiectasis (8%). After respiratory tract manifestations, the skin was the organ most affected. Ten patients (77%) developed multiple skin abscesses on the face, neck, and limbs and to some lesser extent skin rashes, ecthyma gangrenosum, and pustules. Other deep seeded abscesses include liver abscesses in 2 patients (15%) one of whom had 2 episodes, lymph node abscesses in 2 patients (15%) and lung abscess in one patient (8%).

Lymph node manifestations were observed in 8 patients (62%), of which 6 patients had multiple episodes of lymphadenopathy. Meningitis affected 4 patients (31%), of which one patient developed multiple episodes. Septicaemia was reported in 5 (38%) patients although the causative pathogen was not identified in the majority.

BCG vaccine was administered at birth to 11 patients (85%) in the group except for P-01 and P-12 (siblings) owing to their family history of infant deaths. Two patients developed adverse reactions to the vaccine. For instance, P-03 developed an adverse reaction at the BCG scar site along with left axillary lymphadenopathy and a positive Mantoux test, suggestive of possible infection with *M. bovis.* Furthermore, mediastinal masses with caseous necrosis was seen in two patients (P-05 and P-08) indicating disseminated mycobacterial infections. Two patients were diagnosed with tuberculous meningitis, one of whom had a positive Mantoux test (26 mm). Ten (77%) patients received anti-TB therapy despite not being able to isolate the organism.

The patients were commenced on prophylaxis antimicrobials; mainly itraconazole and cotrimoxazole, without delay upon their diagnosis. However, 5 (38%) patients developed multiple infections and manifestations such as LRTI, lymphadenopathy, skin abscesses despite prophylactic treatment. Interferon gamma therapy is not offered in Sri Lanka. One patient (P-02) who underwent stem cells transplantation died following the procedure.

### Mutation analysis

**Fig. 1:**
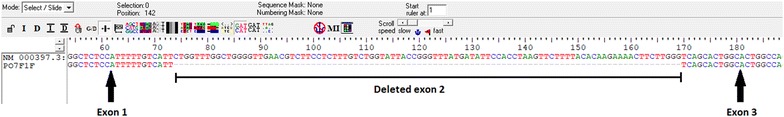
Exon skipping in P-07. The novel splice site mutation identified resulted in the deletion of the entire exon 2 region of the *CYBB* cDNA in P-07. The figure shows the deleted region when the patient cDNA (bottom) is aligned with a reference sequence (top) (NCBI Accession: NC_000023.9)

Eight CGD patients (7 males and 1 female) who were laboratory confirmed by slide NBT assay were included in the mutation analysis. Point mutations in *CYBB* gene were identified in 06 patients confirming their X-linked CGD status. The detected mutations in *CYBB* gene includes, deletions, nonsense and a novel splice site mutation as summarized in Table 2. In P-07 no exonic mutations were identified. However, a novel T > A nucleotide substitution (c.141+6T > A) was detected 6 bases downstream from end of exon 2 in the intron 2 region. Analysis of the patient’s cDNA revealed that this novel mutation is a pathogenic splice site mutation, which resulted in exon skipping by the deletion of the entire exon 2 (Fig. 1).

**Table 2:** Summary of clinical details and genetic analysis of CGD patients

Patient	CGD subtype	Slide NBT result (%)	Age at onset	Age at diagnosis	Gene affected	Exon/intron	Nucleotide change	Amino acid change [9]
P-01	X-linked	< 5	Referred at birth	1 month	*CYBB*	Exon 10	Deletion c.1314delG	p.Ile439SerfsX63
P-02	X-linked	< 5	1.5 months	6.5 months	*CYBB*	Exon 11	Deletion c.1415delG	p.Gly472AlafsX30
P-03	X-linked	< 5	1 month	7 months	*CYBB*	Exon 10	Deletion c.1314delG	p.Ile439SerfsX63
P-04	X-linked	< 5	16 days	2 months	*CYBB*	Exon 3	Nonsense c.217C > T	p.Arg73X
P-05	AR	< 5	6 months	2.5 years	*NCF1*	Exon 2	Deletion c.75_76delGT	p.Tyr26Hisfs
P-06	AR	< 5	1.6 years	10 years	*NCF1*	Exon 2	Deletion c.75_76delGT	p.Tyr26Hisfs
P-07	X-linked	1–2	1 month	2.5 months	*CYBB*	Intron 2	Splice site c.141 + 6T > A	del exon 2 p.Leu16_Gly47del
P-13	X-linked	< 5	4 months	7 months	*CYBB*	Exon 3	Splice site c.252G > A	del. exon 3 p.Ser48_Ala84del

The two remaining patients (P-05 and P-06) were classified as AR-CGD cases, based on the detection of the prominent homozygous GT deletion (c.75_76delGT) mutation in *NCF1* gene.

## Discussion

The majority of the Sri Lankan patients studied, were X-linked CGD cases (75%) similar to other studies in Western countries [1, 10, 11] and in contrast to South Asian (Indian) and other Asian countries such as Turkey and Iran, where AR-CGD is reported with a higher frequency than X-linked CGD [3, 12] (Table 3). Although consanguineous marriages are responsible for this high rates of AR-CGD in Turkey and Iran, consanguinity was not frequent among the Indian patients (2/17), and the higher prevalence of AR-CGD may be due to high incidence of autosomal recessive mutations resulting from marriages being restricted to close-knit communities [2]. Interestingly, 6 patients from 4 families reported to have consanguineous parents in the Sri Lankan cohort, although only 2 patients were positively confirmed with AR-CGD.

**Table 3:** Comparison of patient cohort demographics of different CGD studies

Country/region	Number of patients	Gender		Consangui-nity	CGD subtype		Deaths
		Male	Female		X-linked	AR	
Sri Lanka	12	10	2	6 (46%)	6 (75%)	2 (25%)	4 (38.4%)
India [2]	17	15	2	2 (11.7%)	7 (41%)	10 (59%)	6 (35%)
Iran [12]	41	29	12	23 (56.1%)			5 (12.2%)
Turkey [3]	89	64	25	42 (57.5%)	34 (38.2%)	55 (61.8%)	9 (10.1%)
China [14]	48	44	4	0	36 (75%)	3 (6%)	11 (22%)
Latin America [10]	71	58	13	–	53 (74.6%)	18 (25.3%)	–
Europe [1]	429	351	78	–	290 (67%)	139 (33%)	84 (20%)
USA [11]	368	316	52	–	259 (70%)	81 (22%)	65 (17.6%)

The overall mortality (38%) was higher than in India (35%), and in the West [1, 2]. The mortality in a large cohort of 429 patients from Europe was 20% [1], whereas in an Italian cohort the mortality was 13% [13].

The onset of symptoms in the patient cohort was very early (mean 5½ months) compared with other studies; 20 months in India [2] and 23.9 months in Latin America [10]. Majority of the CGD patients (85%) developed at least one unusual or severe infection within the 1st year of life. Seventy seven percent of the local cohort was identified by unusual susceptibility to severe infections before the age of 2 years. In China 90% of the patients had an onset within 3 months of birth [14]. The mean age at diagnosis of Sri Lankan patients was 1.6 years. This is lower than other countries; India 4½ years [2], Iran 6.3 years [12], Turkey 4.2 years [3], China 2.24 years [14], Europe 4.9 years (X-linked), 8.8 years (AR) [1]. This may be due to early exposure to infections, genetic phenotype, or easy access to basic healthcare in the country. Additionally, the mean diagnostic delay in local patients was about 15 months, which is significantly lower than the 3.9 year delay in Iran [12].

Large scale CGD studies have shown that the disease manifests earlier in X-linked CGD compared to AR-CGD [1]. Similarly, the mean age of diagnosis is lower in the patients with X-linked CGD compared with those with AR-CGD. For instance, the mean age at diagnosis in patients with X-linked CGD versus AR-CGD are 4.9 vs 8.8 years in Europe, 3.0 vs 7.8 years in USA, and 2.7 vs 5.2 years in Turkey [1, 3, 11]. The mean age at diagnosis in our study was 4.6 months for X-linked CGD while it was 4 years and 3 months for AR-CGD. Despite the small size of the cohort in Sri Lanka, the onset of disease was early (1.5 months) in X-linked CGD patients compared to AR-CGD patients (12 months). All of these findings suggest a milder phenotype or a delay in clinical presentation in AR-CGD.

The clinical presentation and manifestations in the local cohort appear to be comparable with regional and global trends. Most large cohort studies report pulmonary manifestations to be the most common (Table 4). In the local cohort 85% had lower respiratory tract infections. Seventy seven percent of the patients had skin/subcutaneous infections while 62% reported lymph node involvement. These figures seem to correlate with other worldwide figures [1, 3, 10]. Gastrointestinal manifestations such as inflammatory bowel disease were not seen in our patients. In addition, chronic lung disease, including bronchiectasis and fibrosis was seen in only one patient. This is in contrast to other cohorts, where it is more common [15].

**Table 4:** Comparison of clinical manifestations in CGD patients

Site of clinical manifestation	Prevalence in different countries/regions—proportion (percentage) (%)							
	Sri Lanka	India [2]	Iran [12]	Turkey [3]	China [14]	Latin America [10]	Europe [1]	USA [11]
Lung	*85*	*82*	66	*83*	*77*	*77*	*66*	*79*
Pneumonia	81							
Pulmonary TB	18							
Lung abscess	9							
Lung fibrosis/bronchiectasis	27							
Skin/subcutis	77	47	63	45	46	42	53	42
Lymph nodes	62	*82*	*76*	65	50	59	50	53
Liver	46	59	–	63	8	–	32	27
Liver abscess	33							
Hepatosplenomegaly	83							
GI tract	31	35	56	22	54	42	48	–
Diarrhoea/dysentery	50							
Oral ulcers	25							
Parotitis	25							
Urinary tract	23	6	–	22	2	20	22	10
Septicaemia	38	25	–	30	23	23	20	18
Ear	8	12	24	22	8	29	14	–
Osteomyelitis	8	18	29	23	4	16	13	25
Meningitis	31	–	2	17	4	–	7	4
TB meningitis	25							
BCG complications	8	–	17	22	53	30	8	–

The most common manifestations reported in each cohort are indicated in italic text

The spectrum of microorganisms implicated in causing infections may vary slightly in different regions, but the most common pathogens reported worldwide are *Staphylococcus aureus*, *Burkholderia cepacia*, *Serratia marcescens*, *Nocardia,* and *Aspergillus* sp. [16]. The Indian study reports *Aspergillus* to be the most common organism isolated in patients followed by *Staphylococcus aureus, Burkholderia cepacia* and *Candida* [2]. However, in Sri Lanka, the causative agent had not been isolated in majority of instances. Due to lack of facilities, bronchoscopy or direct lung biopsies are not routinely done. The patients were often managed in peripheral hospitals, with no input from an Immunologist. Many of the infectious complications occurred before an Immunological diagnosis. *B. cepacia* and *Candida* were isolated from blood in P-04. *Candida* was also isolated from an oral swab two patients with oral thrush, while, septicaemia caused by *Pseudomonas* was reported in P-06. *Pseudomonas aerugenosa* was isolated from a wound swab from P-13. *Aspergillus* sp. was the main pathogen responsible for pulmonary infections in CGD patients in Europe, France, Turkey, and India [1–3, 17]. The lack of reported *Aspergillus* infections in the Sri Lankan cohort is surprising and highly unlikely, suggesting more thorough microbiological examinations are required.

The clinical data collected in the Asian Primary Immunodeficiency (APID) network database suggest that *Mycobacteria tuberculosis* and *M. bovis* infections are significant in Southeast Asian CGD cohorts [18]. Furthermore, a retrospective study on CGD patients with mycobacterial disease from Latin America, Africa, Europe, and Asia, revealed that 44% had TB [19]. In the Chinese cohort of 17 patients, 7 had TB while 8 had BCG complications [20]. Pulmonary tuberculosis was recorded as the second common respiratory involvement (31.7%) in the Iranian cohort [12]. In the Indian cohort, *Mycobacterium* was detected in only one patient, while two more were treated for TB [2].

In Sri Lanka, ten (77%) CGD patients received anti-TB medication at some point in their treatment. The decisions to treat were based on biopsy findings in 5 patients, Mantoux positivity in 3 patients, and X-ray evidence in 2 patients. Facilities for AFB staining, Mantoux, culture, PCR and T spot (IGRA) are available in Sri Lanka, but are not routinely done. Using the acid fast stain on biopsies is generally not done, unless specially requested. Culture is generally done with sputum, but not generally from tissues. In addition, differentiation of *M. bovis* from *M tuberculosis* is not done. PCR (GeneXpert) is performed, but not done routinely due to the cost. On a few occasions PCR and culture were performed. Most often, it was a clinical diagnosis based on BCG immunization, location of lymphadenopathy, presence of necrosis/caseation, and Mantoux (> 10 mm). Interestingly, none of the patients were positive for *Mycobacterium* cultures. TB PCR and acid fast staining were negative. BCG vaccine is given to all infants at birth, and the coverage is 99% in the country [21]. In our cohort, BCG vaccine was given to 11/13 patients. While facilities are available for categorizing BCG infection as definitive, probable, possible, and ruled out (European Society for Immunodeficiencies—ESID criteria) [22], it was not availed of in all instances. In many instances, the diagnosis of BCG/mycobacterial infection was done prior to referral to our clinic. With the clinical history, it is probable that the positive Mantoux was due to infection with *M. bovis*. The diagnosis would fit the possible category (ESID criteria). The BCG strain used the vaccine in Sri Lanka is sensitive to all drugs except pyrazinamide. As the 4 drugs are given in one capsule, pyrazinamide is also administered. The duration for anti TB therapy was the standard regime. ESID suggests 3 drugs for BCG it is in patients with SCID, and continuation till complete immunological reconstitution occurs after HSCT (in SCID for example). For BCGosis, anti-TB treatment including four or more anti-TB drugs should be given until the patient fully recovers, followed by two drugs complete immunological reconstitution after HSCT is achieved. While there are no guidelines for CGD, prolonged therapy was not given. This may have been responsible for the increased mortality.

Genetic analysis of eight Sri Lankan CGD patients revealed that two siblings (P-05 and P-06) had p47*phox* AR-CGD resulting from the prominent GT deletion in exon 2 of *NCF1* gene. The remaining six patients were classified as X-linked CGD. Five patients had previously described [9] *CYBB* mutations. The splice site mutation (c.141+6T > A) identified in the remaining patient (P-07) was a novel mutation. Splice site mutations have been known to be responsible for many genetic diseases including β-thalassemia and CGD [23]. Mutations located in both donor and acceptor splice sites may results in exon skipping and shortened polypeptides. Splice site mutations appear to be a common cause of X-linked CGD with 17.6% of 681 reported mutations falling into this category [9].

The investigators encountered several limitations during the study which are worth mentioning. The Immunodeficiency Clinic at the MRI in Colombo is the sole clinic of its kind to diagnose CGD and other immunological disorders. Patients are referred from across the country to be investigated and diagnosed. The authors believe the prevalence of CGD in the country could be higher, and that a percentage of patients remain unreported and undiagnosed. Paucity of knowledge about primary immune deficiency among medical staff is one problem. Lack of resources to investigate and accurately diagnose infectious pathogens especially BCG disease and TB has contributed to the high mortality rate.

The CGD diagnosis in this study was based on the qualitative slide NBT assay. This assay is now being replaced by the flow cytometry based Dihydrorodemine (DHR) assay as the slide NBT assay is prone to observer bias and requires expertise. The DHR assay has not yet been established and validated in the country due to lack of resources. Furthermore, only eight patients of the cohort were sequenced. Four out of the five remaining patients could not be sequenced as they were already deceased by the time the genetic assay was established and validated, whereas the remaining patient is yet to be sequenced.

## Conclusions

The Sri Lankan CGD patient cohort displayed classic clinical manifestation associated with CGD with pulmonary infections being the most common. Patients suffered recurrent severe infection despite using anti-microbial prophylaxis. Mycobacterial infections are potentially lethal in CGD patients and BCG vaccination should be avoided in infants with the disease. Despite the low number of patients to draw definite conclusions, mortality rate in Sri Lankan cohort remains high. The onset of disease and age at diagnosis is low in comparison to regional countries. Furthermore, the findings support the notion that X-linked CGD manifests earlier and is more severe than AR-CGD. An important highlight of the study was the identification of a novel *CYBB* pathogenic mutation.
